# Advances in gold and silver nanoplatforms for photon therapies in breast cancer

**DOI:** 10.1007/s12551-026-01459-2

**Published:** 2026-09-11

**Authors:** Ana Lívia F. Santos, Camila A. Salvego, Camila R. Silva, Jamerson A. S. Honorio, Joselin J. M. Martinez, Suzana L. Silva, Martha S. Ribeiro, Adriana Fontes

**Affiliations:** 1 Department of Biophysics and Radiobiology, Federal University of Pernambuco (UFPE), Recife, PE 50670-901 Brazil; 2 Department of Biochemistry, Federal University of Paraná (UFPR), Curitiba, PR Brazil; 3 Center for Lasers and Applications, Nuclear and Energy Research Institute (IPEN-CNEN), São Paulo, SP 05508-000 Brazil

**Keywords:** Breast tumor cells, Ionizing radiation, Metallic nanoparticles, Optical radiation, Photodynamic therapy, Photothermal therapy, Radiotherapy

## Abstract

Breast cancer (BC) remains a major global health challenge due to tumor heterogeneity, therapeutic resistance, recurrence, and treatment-associated toxicity. Photon-based therapies, including photodynamic therapy (PDT), photothermal therapy (PTT), and radiotherapy (RT), offer spatially controlled approaches for cancer treatment. However, their efficacy can be limited by insufficient selectivity, restricted light penetration, hypoxia, and radioresistance. Metallic nanoparticles (NPs), particularly gold (AuNPs) and silver nanoparticles (AgNPs), have emerged as versatile platforms capable of enhancing photon interactions across the electromagnetic spectrum. This review discusses recent advances in AuNP- and AgNP-assisted photon therapies for BC, emphasizing how NP size, shape, surface functionalization, optical properties, and biological interactions can influence outcomes. In PTT, plasmonic nanostructures enable efficient light-to-heat conversion, whereas in PDT, metallic NPs can improve photosensitizer delivery, modulate reactive oxygen species generation, and may enhance responses through plasmonic effects. In RT, high-atomic-number nanomaterials increase local energy deposition and contribute to radiosensitization through physical and biological mechanisms. Emerging bimodal approaches integrating PDT, PTT, and RT into a single nanoplatform are also discussed as strategies to amplify antitumor responses. Future perspectives highlight next-generation Au and Ag nanoplatforms, including matrix-based delivery systems, hybrid and asymmetric architectures, nanozyme-integrated approaches, and theranostic strategies designed to improve tumor selectivity and clinical translation. Despite encouraging preclinical results, clinical translation remains challenged by variability in NP design and incomplete standardization of irradiation parameters. Overall, Au and Ag nanoplatforms represent promising tools for advancing precision photon-based therapies, although further optimization and rigorous translational studies are required to establish their clinical potential in BC management.

## Introduction

Breast cancer (BC) remains the most frequently diagnosed cancer among women worldwide and one of the leading causes of cancer-related mortality (WHO, 2026). Despite major advances in early detection and multimodal treatment, including surgery, systemic therapies (chemo-, hormonal, and immuno-approaches), and radiotherapy (RT), significant clinical challenges persist, such as therapeutic resistance, local recurrence, metastatic dissemination, and treatment-associated adverse effects. Moreover, the marked biological heterogeneity of BC, encompassing hormone receptor-positive, anti-human epidermal growth factor receptor 2 (HER2)-positive, and triple-negative BC subtypes, often results in variable therapeutic responses and distinct prognostic outcomes (Day et al. 2026). These limitations highlight the need for more precise, selective, and adaptable therapeutic strategies.

The therapeutic application of electromagnetic radiation, encompassing both non-ionizing and ionizing regions of the spectrum, has significantly advanced modern medicine by enabling precise and tissue-sparing treatments for a wide array of diseases. Modalities based on visible and near-infrared (NIR) light, used in phototherapies like photodynamic therapy (PDT) and photothermal therapy (PTT) (Liang et al. 2025; Lovras et al. 2025), as well as ionizing radiation used in RT (Al Balushi and King 2025; Deng and Zhu 2026), play a central role in contemporary oncology. Although these approaches interact with tumor tissues through distinct physical mechanisms, they share a common objective of delivering energy with spatial precision to eradicate malignant cells while minimizing collateral injury to surrounding healthy tissues. Nevertheless, several limitations persist, including restricted tissue penetration at optical wavelengths, inadequate tumor selectivity, intratumoral hypoxia, radioresistance, tumor heterogeneity, and dose-limiting toxicity.

Nanotechnology offers a promising approach to overcoming many of these challenges. Among the nanomaterials explored for biomedical use, metallic nanoparticles (NPs) have attracted significant attention because of their distinctive physicochemical and optical properties. Gold (Au) and silver (Ag) NPs, in particular, can serve as versatile platforms for efficient photon interactions across diverse energy ranges, mediated by their tunable size and shape. Their adjustable surface properties, including charge and functional coatings, can also modulate biodistribution and cellular uptake, enhancing therapeutic outcomes. Additionally, these materials can be engineered to deliver drugs, photosensitizers (PSs), targeting ligands, or imaging agents, thereby enabling multifunctional systems with both diagnostic and therapeutic functions (Dhir et al. 2024).

An important consideration in using NPs for cancer therapy is their biodistribution and ability to accumulate within tumor tissues. Passive tumor accumulation has historically been associated with the enhanced permeability and retention (EPR) effect, in which abnormal tumor vasculature, characterized by leaky blood vessels and impaired lymphatic drainage, can facilitate the extravasation and retention of nanoscale materials within the tumor microenvironment (TME) (Maeda 2012). Although the magnitude of the EPR effect varies among tumor types and between experimental models and patients, it has historically underpinned the passive targeting of nanomedicines and has motivated the development of NP-based platforms for cancer diagnosis and therapy (Nichols and Bae 2014). Surface functionalization with targeting ligands can further enhance tumor localization and cellular uptake, complementing passive accumulation mechanisms (Danhier et al. 2010). However, the in vivo biodistribution and tumor accumulation of metal-based NPs remain highly variable and depend on their physicochemical properties, surface characteristics, route of administration, and the TME. NPs may also accumulate in off-target organs, particularly the liver and spleen following intravenous administration (Chen et al. 2023). In BC, NP accumulation is influenced by multiple TME characteristics, including vascular permeability, extracellular matrix composition, interstitial fluid pressure, and molecular subtype, which can modulate the magnitude of the EPR effect (Sindhwani et al. 2020).

Once NPs reach the tumor and interact with cancer cells, their size, surface charge, and functionalization influence cellular uptake. Smaller NPs may be internalized more readily, although this depends on their physicochemical properties and cell type, while larger NPs may remain predominantly extracellular. Non-functionalized NPs are generally internalized through non-specific endocytosis, whereas functionalization can promote receptor- or transporter-mediated uptake. After internalization, NPs traffic through endosomal and lysosomal compartments, with persistence and localization depending on NP properties and cellular context (Sell et al. 2023; Toscano and Torres-Arias 2023).

Metallic NPs have been widely studied for their exceptional optical properties arising from localized surface plasmon resonance (LSPR) (Aminabad et al. 2019). When exposed to appropriate wavelengths, metallic nanostructures can efficiently convert absorbed light into heat, enabling effective photothermal effects (Bucharskaya et al. 2022). Furthermore, metallic NPs, especially AuNPs and AgNPs, can enhance photodynamic processes by facilitating PS delivery, improving PS excitation, and boosting reactive oxygen species (ROS) generation under light exposure (Lockwood et al. 2025). Notably, AuNPs also exhibit high chemical stability. The utility of AuNPs and AgNPs also extends to ionizing radiation, as their high atomic number promotes photoelectric interactions and secondary electron emission, thereby increasing local dose deposition and enhancing radiosensitization (Mateo et al. 2026). AuNPs have been used in particular in this context due to their strong radiation–matter interactions. Ag-based nanosystems have also been explored as radiosensitizers via mechanisms involving oxidative stress, which can trigger autophagy (Liu et al. 2018; Wu et al. 2016). These attributes position Au and Ag nanomaterials as highly promising therapeutic agents.

The integration of AuNPs and AgNPs with photon-based therapies exemplifies the broader movement toward precision medicine, where treatment efficacy is determined by both the radiation source and the controlled modulation of the local biological microenvironment. By focusing energy deposition at diseased sites, enhancing intracellular delivery, overcoming resistance mechanisms, and enabling multimodal approaches, these metallic nanomaterials have the potential to significantly improve therapeutic outcomes. These advancements are especially pertinent in the management of BC.

This review examines the current landscape of NPs with cores composed of Au, Ag, or Au–Ag, which serve as versatile agents in photon-based therapies across the electromagnetic spectrum, encompassing both non-ionizing optical modalities and X-ray- or gamma-based applications. Special attention is given to recent advances in Au- and Ag-based nanoplatforms for BC treatment, with a focus on how NP design, surface engineering, and biological interactions influence therapeutic outcomes. In addition, emerging directions in next-generation nanosystems are discussed, including matrix-based delivery approaches, hybrid and asymmetric architectures, nanozyme-assisted strategies, and theranostic platforms integrating therapeutic and diagnostic functionalities. By bridging current preclinical advances and future technological perspectives, this review aims to provide a comprehensive view of the opportunities and challenges in translating metallic nanoparticle-assisted photon therapies into clinically relevant strategies for BC management.

## Au and Ag nanoparticles in phototherapy

### Basic principles

The optical response of metallic NPs can be adjusted by controlling parameters such as size, shape, and composition, as well as the surrounding dielectric environment, which directly influences the spectral region of their extinction (absorption plus scattering) bands. These nanomaterials can adopt various geometries, including spherical, rod-like, and pyramidal shapes (Aminzai et al. 2024; Kelly et al. 2003).

When the incident light wavelength matches the extinction band, electrons in the conduction band undergo collective oscillations driven by the electromagnetic field, giving rise to LSPR. For this reason, the extinction band is also called the plasmon band. Nanostructures based on Au and Ag, as well as their Au–Ag combinations, can exhibit plasmon bands covering a wide portion of the visible and NIR spectrum (Kelly et al. 2003; Rodrigues et al. 2024; Zahdeh and Karaman 2025). In particular, plasmon bands in the NIR region can be advantageous for biological applications, as the NIR enables deeper tissue penetration (Pansare et al. 2012).

**Fig. 1:**
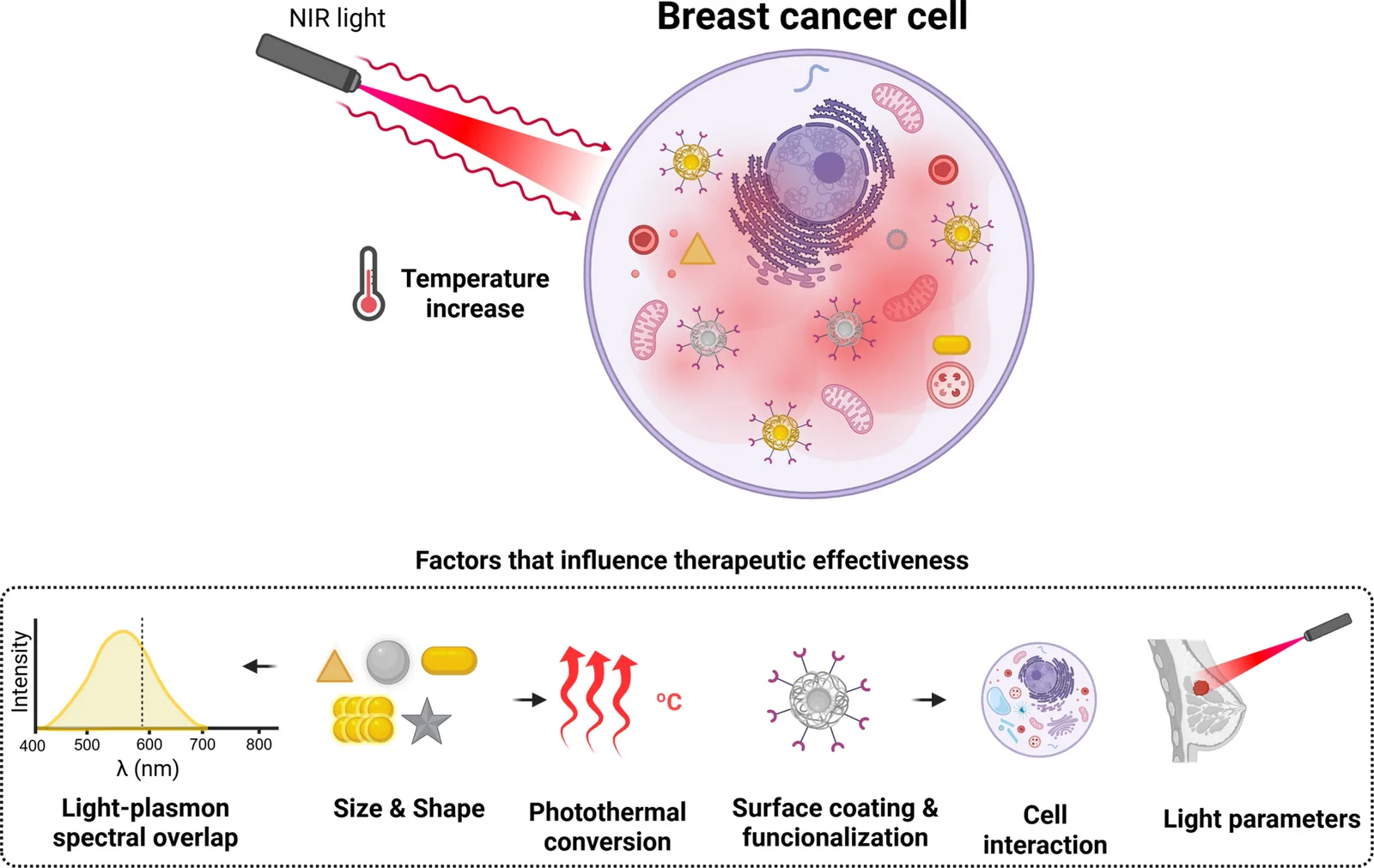
Schematic representation of PTT mediated by metallic NPs in BC cells. Upon irradiation with light at wavelengths matching the LSPR of the NPs, absorbed optical energy is converted into heat, resulting in localized hyperthermia and subsequent tumor cell damage. The photothermal efficiency of the nanosystem is influenced by physicochemical and biological factors, including the overlap between the excitation wavelength and the plasmon resonance band, NP size and shape, photothermal conversion efficiency, surface coating and functionalization, cellular interactions and uptake, and irradiation parameters such as wavelength, irradiance, and exposure time. These factors jointly determine heat generation, intracellular responses, and the overall therapeutic outcome of PTT. Created with BioRender.com, agreement number: NH29SD2YMH

The LSPR effect can convert light energy into heat, which is why Au, Ag, and Au–Ag NPs have been widely explored in PTT (Fig. 1). The photothermal effect arises from the non-radiative decay of the LSPR, in which the absorbed optical energy is converted into heat via electron–electron and electron–phonon relaxation processes (Ajib et al. 2026; Skinner et al. 2025). In this regard, anisotropic nanostructures such as gold nanorods (AuNRs) are interesting for PTT, as their aspect ratio-dependent longitudinal plasmon mode enables strong and tunable absorption in the NIR region and concentrates electromagnetic energy along the long axis, generating intense local fields and promoting efficient non-radiative relaxation into heat (Skinner et al. 2025).

**Fig. 2:**
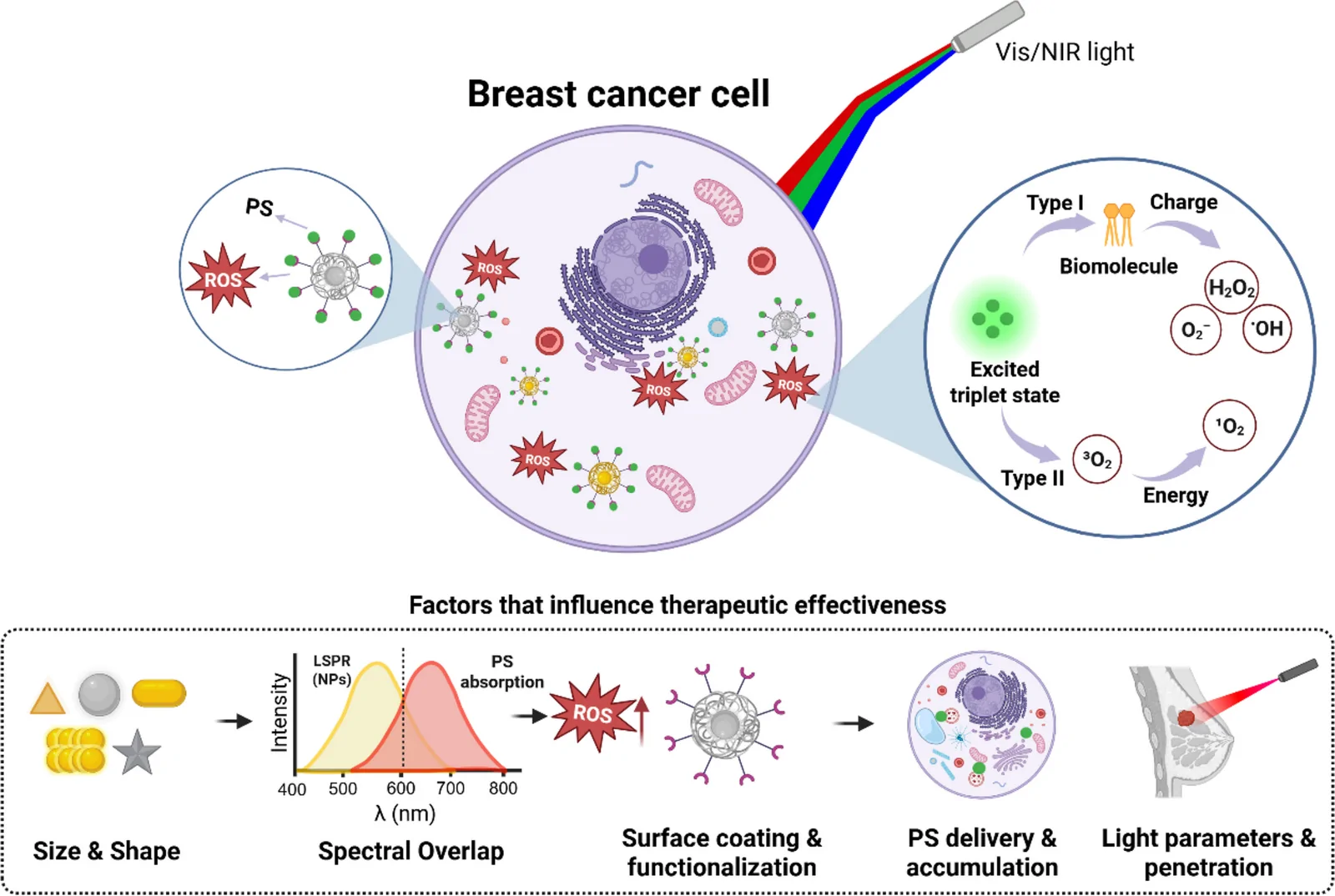
Schematic representation of PDT mediated by metallic NPs in BC cells. Following light irradiation, PSs associated with metallic NPs are excited from the ground state to an excited singlet state and subsequently undergo intersystem crossing to the triplet state. The excited triplet PS can then generate ROS through Type I reactions, involving electron or hydrogen transfer (*e.g.*, O_2_^−^, H_2_O_2_, •OH), and/or Type II reactions, involving energy transfer to molecular oxygen, producing singlet oxygen (^1^O_2_). The resulting oxidative stress damages cellular components and promotes tumor cell death. The therapeutic performance of NP-assisted PDT is influenced by multiple factors, including NP size and shape, spectral overlap between the NP plasmon band and the PS absorption spectrum, surface coating and functionalization, PS delivery and intracellular uptake, light parameters, and tissue penetration. These factors collectively determine the therapeutic effect. Created with BioRender.com, agreement number: WR29SD2N1I

Beyond their photothermal properties, metallic NPs can also be associated with PSs to improve photodynamic efficiency (Fig. 2). These NPs can act as carriers for PSs, thereby improving their delivery and cellular interactions (Ajib et al. 2026; Da Silva et al. 2025). Moreover, the tunability of the plasmon band makes them attractive for optimizing the interaction between PSs and light. For instance, LSPR can intensify the electromagnetic field near the NP, thereby enhancing PS excitation and boosting ROS generation. Facilitating NP–PS energy (or electron) transfer processes can also contribute to intensification, depending on the nanosystem’s physicochemical configuration. For this to occur effectively, the PS absorption spectrum must overlap with the NP plasmon band, with a short separation between them (Rodrigues et al. 2024; Tada and Baptista 2015). Nevertheless, a complete understanding of LSPR’s contribution to enhancing photodynamic effects is still under investigation.

NP size also influences the balance between absorption and scattering: smaller nanostructures favor absorption-dominated extinction, enhancing photothermal conversion efficiency for PTT, whereas larger ones exhibit stronger scattering that can modulate the local electromagnetic field distribution at the nanoscale, thereby influencing photodynamic responses (Kim et al. 2019). Moreover, the affinity of AuNPs and AgNPs for thiol-containing molecules can be particularly advantageous for assisting targeted delivery of nanosystems for therapeutic purposes. NP functionalization can also confer surface charges, enhance cell interaction, improve colloidal stability in biological environments, and decrease cytotoxicity (Hlapisi et al. 2024; Pasparakis 2022).

In general, these NPs can be relatively easily synthesized in aqueous media, with chemical routes based on the reduction of metal salts being more commonly employed for biological applications. The reducing agents may include conventional chemicals, natural compounds, or even microorganisms (Ajib et al. 2026; Aminzai et al. 2024).

Together, these properties make AuNPs and AgNPs attractive platforms for phototherapies in BC research, as will be presented in the following sections.

### Applications in photothermal therapy

Spherical AuNPs remain among the most widely investigated plasmonic nanostructures for PTT due to their relatively simple synthesis, tunable optical properties, and efficient photothermal conversion, with LSPR bands in the green region of the visible spectrum (520–550 nm). Recent studies have explored these AuNPs against BC cells (Table 1).

**Table 1:** Summary of AuNP- and AgNP-mediated PTT approaches in BC models

NP type	Size (nm)	Plasmon peak (nm)	Coating/functionalization	Experimental model	Light parameters				NP concentration	Main outcomes	Ref
					***L*** (nm)	Irradiance (mW/cm^2^)	Light dose (J/cm^2^)	Time (s)			
Au nanorods	10.3 ± 1.0 × 44.0 ± 4.3 (TEM)	808 (longitudinal peak)	-	In vitro 4T1	808 (laser)	4500	NI	300	1.5 to 120 µg/mL	AuNPs synthesized using MUTAB as a reducing agent did not cause toxicity in doses lower than 6 µg/mL in dark conditions. Following irradiation, 1.5, 3, and 6 µg/mL induced decreases of 60%, 79%, and 94% in cell viability, respectively	Bianchi et al. (2024)
Au nanospheres	14.6 ± 1.4 (TEM)	526	Silica	In vitro MDA-MB-231 and MCF-7	525 (LED)	4.8 ± 0.2	NI	1200	1 × 10^12^ NPs/mL	Delivery mediated by nanostraws induced a better PTT performance, with cell viability decreased to 34.6% for MDA-MB-231 and 29.8% for MCF-7	Camacho et al. (2025)
Au nanospheres	22.15 (AuCh-NPs) and 12 (AuCit-NPs) (TEM)	520 (AuCh-NPs) 525 (AuCit-NPs)	-	In vitro MCF-7	530 (LED)	181	325.8 and 488.7	1800 and 2700	2, 4, 6, and 8 µM	AuCh-NPs prepared using fungus extract reduced cell viability to 26–28% under irradiation (IC50 = 5.56 µM) after 30 min and promoted higher inhibition of cell migration than AuCit-NPs	Ezzat et al. (2025)
Au nanorods	10 ± 2 × 55 ± 3 (TEM)	800 (longitudinal peak)	Mesoporous silica combined with the 5-azacytidine and mocetinostat	In vitro 4T1 In vivo 4T1 tumor-bearing BALB/c mice	808 (laser)	500 or 300, depending on the assay	NI	300	0.2, 0.5, and 1.0 µM (in vitro) 100 µg/mL (in vivo)	Under irradiation, the nanosystem caused a near-complete loss of cell viability. PTT also led to a tumor inhibition rate of 85.8% and induced immune memory	Gao et al. (2026)
Ag nanotriangles	123.0 ± 13.9 (TEM)	808	PEG combined with PD-L1-targeting aptamers	In vitro MDA-MB-231 In vivo 4T1 tumor-bearing BALB/c mice	808 (laser)	In vitro 2000 In vivo 1000	NI	In vitro 180 In vivo 300	2, 5, 10, and 50 µg/mL	Upon irradiation, for 5 µg/m of apt-NP, low cell proliferation (38.19 ± 4.57%) was observed with apoptosis occurring at an early stage. In vivo*,* after 5 min (5 mg/kg of apt-NP), the growth inhibition rate was 68.73%	Li et al. (2025)
Au nanospheres	50 (TEM)	541	-	In vitro MDA-MB-231	532 (laser)	NI	NI	600	10, 20, 30, 40, 50, 60,70, 100, and 130 µg/mL	Software was used to estimate the optimal NP size and plasmon peak-to-PTT. PTT with 50 µg/mL reduced viability to 61.2%	Mousavifard et al. (2025)
Au nanospheres	77.3 ± 13.4 (AuNPs@BSA) (DLS)	545 (AuNPs@BSA)	BSA	In vitro MDA-MB-231	532 (laser)	NI	NI	300	200, 400, 600, 800, 1000 µg/mL	300 s of irradiation with AuNPs@BSA triggered oxidative stress and induced apoptosis at 800 µg/mL (IC50)	Salabat et al. (2025)
Au quasi-spherical NPs	71.9 ± 0.3 CADA-AuNPs (DLS)	545	-	In vitro MDA-MB-231	808 (laser)	600	NI	300	0 to 160 µg/mL	Under irradiation, cell viability decreased in a concentration-dependent manner, reaching 24.6% at 20 µg/mL	Vashisth et al. (2024)

*Apt* aptamer, *AuCh-NPs* Chaga mushroom-mediated AuNPs, *AuCit-NPs* citrate-mediated AuNPs, *BSA* bovine serum albumin, *CADA* choline decanoate, *DLS* dynamic light scattering, *IC50* half-maximal inhibitory concentration, *MUTAB* (11-mercaptoundecyl)-*N,N,N*-trimethylammonium bromide, *PD-L1* programmed death-ligand 1, *PEG* polyethylene glycol, *TEM* transmission electron microscopy, *NI* not informed

Computational modeling can elegantly establish relationships between absorption cross-sections in the green spectrum and NP size. For spherical AuNPs with diameters near 50 nm, optimal conditions for maximizing photothermal conversion have been identified, with predictions closely matching experimental cytotoxicity outcomes (Mousavifard et al. 2025). Based on these findings, AuNPs were synthesized and incubated with MDA-MB-231 cells (a model of triple-negative BC, TNBC) at concentrations ranging from 10 to 130 µg/mL, then irradiated with a 532 nm Nd:YAG laser for 10 min. MTT assays demonstrated dose-dependent cytotoxicity, with cell viability decreasing to approximately 60%, 40%, and 20% at concentrations of 70, 100, and 130 µg/mL, respectively, corresponding to a half-maximal inhibitory concentration (IC50) of 70 µg/mL. After PTT, viability decreased to 61.2% at 50 µg/mL, accompanied by marked morphological alterations, apoptosis, and cell death.

Achieving selective cytotoxicity while preserving healthy tissue has been a challenge in PTT-based BC treatment. Within this framework, photothermal platforms based on AuNPs functionalized with bovine serum albumin (AuNPs@BSA) can improve biocompatibility and selectively induce apoptosis in MDA-MB-231 cells (Salabat et al. 2025). After BSA functionalization, the plasmon band shifted from 535 to 545 nm, confirming conjugation. Upon irradiation with a 532-nm laser, AuNPs@BSA increased the temperature by approximately 40 °C after 5 min. Furthermore, in vitro assays showed reduced cell viability after 24 h of incubation, depending on the nanosystem concentration, with an IC50 of 800 µg/mL. Following treatment, elevated lactate dehydrogenase activity suggested mitochondrial dysfunction, and apoptosis was confirmed by cytoplasmic shrinkage, reduced cell diameter, and flow cytometry analyses.

Increasing NP internalization and modulating their intracellular localization also play important roles in PTT. In this context, nanostraw-assisted intracellular delivery was shown to enhance the photothermal efficacy of silica-coated AuNPs (AuSHINs) against BC (Camacho et al. 2025). Following synthesis, AuNPs were covered with (3-aminopropyl)trimethoxysilane to form a silica shell, generating core–shell nanostructures with an average core size of ~15 nm and a silica shell thickness of ~6.0 nm, as confirmed by transmission electron microscopy (TEM) and scanning electron microscopy (SEM) analyses. The silica coating altered the refractive index of the surrounding medium, promoting a redshift in the extinction spectrum (from 519 to 526 nm). To evaluate cellular uptake, AuSHINs were conjugated with the fluorophore ATTO 647 N and incubated with MDA-MB-231 and MCF-7 BC cells (luminal-type, a model of hormone-responsive BC) for 0.5 and 2 h, followed by irradiation with a 525 nm green LED for 20 min. Conventional incubation with AuSHINs moderately reduced cell viability, whereas nanostraw-mediated intracellular delivery markedly enhanced photothermal cytotoxicity, reducing viability to 34.6% in MDA-MB-231 cells and 29.8% in MCF-7 cells, demonstrating the importance of increased NP accumulation within cells. Irradiation of AuSHINs increased the temperature by approximately 16 °C, confirming efficient photothermal conversion. Fluorescence microscopy revealed greater NP internalization over time, with most AuSHINs colocalizing with lysosomes after 2 h, suggesting predominantly endocytic uptake and indicating that intracellular NP accumulation may influence the photothermal response. Additionally, treatment with *N*-acetyl-L-cysteine partially restored cell viability, suggesting that ROS generation contributes to the therapeutic effect.

Beyond traditional chemical synthesis using sodium citrate, environmentally sustainable approaches have gained increasing attention for producing biologically active AuNPs with improved biocompatibility. Accordingly, mycosynthesized AuNPs prepared using Chaga mushroom extract (AuCh-NPs) were investigated as a photothermal platform against MCF-7 cells and compared with conventionally synthesized citrate-reduced AuNPs (AuCit-NPs) (Ezzat et al. 2025). UV–Vis spectroscopy revealed plasmon bands at 525 nm for AuCh-NPs and 520 nm for AuCit-NPs, while TEM analyses showed predominantly spherical nanostructures with average diameters of approximately 22 and 12 nm, respectively. Cytotoxicity assays demonstrated that AuCh-NPs exhibited greater activity than AuCit-NPs against MCF-7 cells. Under LED irradiation (530 nm), AuCh-NPs reduced cell viability to approximately 26–28% after 30 min and also promoted the strongest inhibition of cell migration.

In addition to spherical shapes, AuNPs can adopt different morphologies, expanding the spectral range of their plasmon bands. Among these, AuNRs have been explored because their longitudinal LSPR falls in the NIR region, which is advantageous for PTT. A multifunctional nanosystem based on mesoporous silica-coated AuNRs combined with the epigenetic agents 5-azacytidine and mocetinostat, and further coated with macrophage membranes (GS@AM@M), was developed to enhance tumor targeting and immune evasion (Gao et al. 2026). TEM analyses showed AuNRs approximately 55 nm in length and 10 nm in diameter, surrounded by a mesoporous silica shell ~23 nm thick. In vitro assays using murine 4T1 triple-negative breast cancer (TNBC) cells demonstrated enhanced NP internalization and minimal dark cytotoxicity. However, after 808 nm irradiation for 5 min, the nanosystem induced pronounced photothermal cytotoxicity, reducing cell viability to below 50% at 0.5 µM and nearly eliminating viable cells at 2 µM, while also suppressing cellular proliferation. In vivo studies in BALB/c mice revealed rapid temperature increases at the tumor site, reaching approximately 55 °C after irradiation, resulting in an 85.8% tumor inhibition rate for GS@AM@M when the nanosystem was administered 5× via intravenous injection every 3 days and irradiated 3×. Mechanistically, the treatment promoted immunogenic cell death by suppressing MYC oncogene expression, thereby enhancing interferon signaling and antitumor immune responses. Furthermore, treated animals developed protective immunity after tumor rechallenge, highlighting the potential of combining PTT with immunomodulatory strategies for TNBC cells.

Because cetyltrimethylammonium bromide (CTAB), commonly used in AuNR synthesis, is cytotoxic, alternative surface coatings have been explored to improve biocompatibility, such as 11-mercaptoundecyl)-*N,N,N*-trimethylammonium bromide (MUTAB) (Bianchi et al. 2024). TEM analyses revealed rod-shaped nanostructures with an average diameter of ~10 nm and length of ~44 nm, while UV-Vis spectroscopy showed a plasmon band centered at 808 nm. In vitro assays using 4T1 cells demonstrated efficient NP internalization after 24 h of incubation. In the dark, cell viability decreased significantly only at concentrations above 12 µg/mL after 48 h of incubation. Upon irradiation with an 808-nm laser for 5 min, AuNRs induced concentration-dependent temperature increases up to 55 °C (6 µg/mL), resulting in marked reductions in cell viability of up to 94% after 24 h of incubation. Additional analyses indicated that treatment predominantly induced late apoptosis or secondary necrosis, highlighting the potential of MUTAB-coated AuNRs for PTT.

In a similar context, biocompatible ionic liquids (ILs) have also been explored for preparing anisotropic AuNPs (Vashisth et al. 2024). Using choline decanoate (CADA) IL in the synthesis, the amount of CTAB required to produce anisotropic AuNPs was significantly reduced. According to the authors, choline-based ILs are particularly interesting because choline plays a role in the human nervous system and metabolism, supporting biomolecule synthesis and cellular structure. These quasi-spherical AuNPs exhibited a plasmon band centered at 545 nm extending into the NIR region and an average size of approximately 72 nm. Due to their optical properties, 808 nm irradiation was used in photothermal experiments. The NPs exhibited a photothermal conversion efficiency of 50.5% and induced a rapid temperature rise of approximately 10 °C within the first 5 min of irradiation. In vitro assays demonstrated high biocompatibility in both MDA-MB-231 BC cells and MCF-10A healthy mammary epithelial cells, in contrast to CTAB-based AuNPs. Under 808 nm irradiation, CADA-AuNPs induced dose-dependent photothermal cytotoxicity in MDA-MB-231 cells, reducing cell viability to 24.6% at 20 µg/mL and 8.1% at 160 µg/mL, with apoptosis identified as the predominant mechanism of cell death.

Beyond AuNPs, AgNPs have also attracted attention for PTT due to their tunable plasmonic properties, particularly when engineered into anisotropic shapes that can shift their plasmon bands into the NIR region (Table 1). From this perspective, PNTs modified with polyethylene glycol (PEG) and conjugated with PD-L1-targeting aptamers (apt-PNTs) were developed for PTT (Li et al. 2025). TEM analyses revealed uniformly dispersed triangular nanostructures with an average edge length of approximately 123 nm, while UV–Vis spectroscopy showed a plasmon band centered at 808 nm. Cellular uptake studies demonstrated maximum NP accumulation after 24 h. Upon 808-nm laser irradiation (3 min, NP concentration of 5 µg/mL), apt-PNTs induced greater photothermal cytotoxicity against MDA-MB-231 cells than non-targeted PNTs, significantly reducing cell proliferation and increasing apoptosis rates. In vivo experiments using 4T1 tumor-bearing mice showed that apt-PNTs (5 mg/kg) under NIR irradiation (10 min) achieved a tumor growth inhibition rate of 68.73%, with tumor temperatures reaching approximately 44 °C after 5 min of laser application.

In summary, these studies highlight the potential of Au and Ag nanostructures for the PTT of BC cells, indicating that NP design, surface modification, and targeted delivery strategies can enhance photothermal efficiency, biocompatibility, cellular interactions, and anticancer activity, while also enabling the use of longer wavelengths to favor deeper light penetration into tissues. Importantly, the therapeutic outcomes achieved by these plasmonic nanoplatforms appear to depend not only on heat generation itself but also on complex biological processes, including intracellular accumulation, subcellular localization, immune modulation, and tumor targeting. Nevertheless, the considerable variability in experimental parameters limits direct comparisons between studies, and most investigations remain restricted to in vitro models. Therefore, despite the promising advances reported so far, further efforts toward protocol standardization, long-term safety evaluation, and optimization of tumor selectivity will be important to support the clinical translation of AuNP- and AgNP-mediated PTT for BC.

### Applications in photodynamic therapy

One of the main applications of AuNPs and AgNPs in PDT is their use as delivery platforms for PSs. In BC models, AuNPs have been investigated for this purpose because of their facile surface functionalization and ability to associate with a wide range of photosensitizing molecules via electrostatic interactions. Recent studies have therefore explored AuNP- and AgNP-based nanosystems designed to optimize PS delivery and improve PDT outcomes (Table 2).

**Table 2:** Summary of AuNP- and AgNP-mediated PDT approaches in BC models

NP type	Size (nm)	Plasmon peak (nm)	Coating/functionalization	Experimental model	Light parameters				PS type	Main outcomes	Ref
					***L*** (nm)	Irradiance (mW/cm^2^)	Light dose (J/cm^2^)	Time (s)			
Ag nanospheres	25 (TEM)	414		In vitro MDA-MB-231	650 (LED)	50	45	900	Sodium copper chlorophyllin (CHL)	AgNPs-CHL significantly decreased the viability of MDA-MB-231 cells, presenting an IC50 of 6.25 µg/mL and reducing viability to below 10% at 50 µg/mL. Upon irradiation, NPs were efficiently internalized into the cytoplasm and induced approximately 60% early apoptosis	Abdel Gaber et al. (2025)
Au nanobipyramids	45 × 80 (TEM)	785	-	In vitro MCF-7	610 (light source not informed)	40	48	1200	Phthalocyanine	No dark toxicity was observed in MCF-7 cells. Upon irradiation, the system exhibited photodynamic effect against the cells at a concentration of 1.7 nM	Cao et al. (2024)
Ag nanospheres	36 ± 7 (TEM)	425	Loaded in liposomes	In vitro MCF-7	660 (laser)	NI	10	NI	Zn phthalocyanine	AgNPs-Lip@ZnPcS_4_ demonstrated high therapeutic efficacy against MCF-7 cells under laser irradiation, achieving the lowest IC50 value (3.7 µg/mL). Cell death occurred via apoptosis with subcellular localization in mitochondria and lysosomes	Chota et al. (2025)
Au core and Ag shell nanospheres	4.79 (B-Au–Ag) 7.49 (1-B-Au–Ag) 9.86 (2-B-Au–Ag) (DLS)	433	PEG and biotin	In vitro MDA-MB-231	625 (LED)	NI	NI	1800	Porphyrins	2-B-Au–Ag showed the highest PDT efficacy, reducing cell viability to 24.67% at 100 µg/mL (compared to 35.21% for 1-B-Au–Ag)	Magaela et al. (2025)
Au nanospheres	2 (TEM)	524	-	In vitro MCF-7	660 (1@AuNPs) 730 (2@AuNPs) (LED)	1.7 1.0	NI	1200	azaBODIPY-1 azaBODIPY-2	1@AuNPs induced the highest photoinhibition (IC50 3.60 ± 0.07 µM) when compared to 2@AuNPs (IC50 10.0 ± 0.05 µM)	Molupe et al. (2026)
Au nanospheres	16 (technique not informed)	520	Galactosyltriazole-PEG_3_-SH	In vitro MDA-MB-231 and SKBR-3	633 (laser)	29	10.5	360	Chlorin e6	Galactose showed higher uptake in both cancer cell lines compared with healthy cells. After irradiation, SKBR-3 cells showed a 48% reduction in viability (50 nM). In MDA-MB-231 cells, no reduction in viability was observed, likely because acidic organelles were destroyed before PDT application	Thomas-Moore et al. (2023)
Au nanospheres	2.5 (TEM)	526	-	In vitro MDA-MB-231	415 (LED)	NI	NI	1800	Porphyrins	AuNPs-InPS induced the highest photocytotoxicity (ca*.* 20% of viable cells). Porphyrins that had sugar ligands (InPS and GaPs) performed better compared to the non-sugar porphyrins	Vukeya et al. (2025)
Au nanospheres	90 (radius) (DLS)	520	Anionic D-g-PAA	In vitro MDA-MB-231	660 (LED)	93.6*	28.1*	600*	Temoporfin	PDT effect against cancer cells (IC50 = 4 µg/mL for nanosystem, containing 0.4 µg/mL temoporfin)	Yeshchenko, et al. (2024)
Au (various shapes)	Mean of 61 ± 7.6 (TEM)	540	Loaded in liposomes	In vitro MDA-MB-231	660 (laser)	NI	10	NI	Pheophorbide-a	The nanocomplex exhibited photocytotoxicity with an IC50 of 10 µg/mL, while PS alone had a lower IC50 (0.3 µg/mL). Cells treated with AuNPs-LIP-PPB-a showed a markedly higher ROS production than when PPB-a was used alone	Zahra et al. (2025)

*AgNPs-CHL* chlorophyllin-functionalized AgNPs, *azaBODIPY* aza-boron-dipyrromethene, *B-Au–Ag* biotin-functionalized Au–AgNPs, *D-g-PAA* dextran-graft-polyacrylamide, *DLS* dynamic light scattering, *GaPS* sugar-functionalized gallium porphyrin, *IC50* half-maximal inhibitory concentration, *InPS* sugar-functionalized indium porphyrin, *LIP* liposome, *PEG* polyethylene glycol, *PPB-a* pheophorbide-a, *ROS* reactive oxygen species, *SH* thiol group, *TEM* transmission electron microscopy, *ZnPcS*_*4*_ zinc phthalocyanine tetrasulfonate, *NI* not informed

^*^600 s was the value informed in the original study, but the calculated value would be 300 s for a light dose of 28.1 J/cm^2^. Considering 600 s of exposure time, the calculated light dose would be 56.2 J/cm^2^. In the case of assuming 600 s and 28.1 J/cm^2^, the calculated irradiance would be 46.8 mW/cm^2^

AzaBODIPYs are boron-dipyrromethene derivatives that exhibit strong absorption in the red and NIR regions, high photostability, and tunable photophysical properties, making them attractive PSs for PDT. However, their hydrophobic nature may limit aqueous dispersibility and cellular delivery, motivating the development of NP-based formulations. Two azaBODIPY PSs (1@AuNP and 2@AuNP) were conjugated to AuNPs via a dative bond to overcome the high hydrophobicity of the free dyes and improve their performance as PDT agents against MCF-7 cells. TEM analysis revealed NP conjugates with diameters ranging from 6 to 10 nm (size of a bare NP, 2 nm). Cellular uptake studies showed greater accumulation of the more hydrophilic azaBODIPY and its AuNP conjugate (2@AuNP). After 24 h of incubation, cells were irradiated for 20 min with LEDs at 660 nm (2.04 J/cm^2^) or 730 nm (1.20 J/cm^2^), according to each PS’s absorption profile. Under irradiation, all formulations induced phototoxic effects that depended on PS concentration. The 1@AuNP conjugate showed the highest PDT efficacy (IC50 = 3.6 µM), markedly improving the activity of the corresponding free dye (IC50 = 11.0 µM). Overall, AuNP conjugation reduced dark toxicity while enhancing photodynamic activity in BC cells (Molupe et al. 2026).

Beyond direct PS–AuNP conjugates, researchers have also investigated hybrid nanosystems that combine metallic NPs with additional delivery vehicles to further improve PS uptake and PDT efficacy. In this context, AuNPs synthesized via a green approach using *Dicoma anomala* leaf extract were incorporated, together with pheophorbide-a (PPB-a), a chlorophyll-derived PS with strong absorption around 660 nm, into liposomes (LIP), thereby generating a multifunctional nanocomplex designed to enhance cellular delivery. TEM and SEM revealed nanostructures with diameters ranging from approximately 66 to 69 nm. After 24 h of incubation, MDA-MB-231 cells were irradiated with a 660-nm laser. The AuNP–LIP–PPB-a nanocomplex induced a concentration-dependent reduction in cell viability. Furthermore, treatment promoted significant mitochondrial membrane depolarization, indicating activation of cell death pathways. Although free PPB-a exhibited greater phototoxicity (IC50 = 0.30 µg/mL), incorporation into the AuNP–LIP platform increased ROS generation (5.5-fold versus 2.5-fold relative to free PPB-a), while maintaining PDT activity (Zahra et al. 2025).

In addition to improving PS delivery, AuNPs can be functionalized with targeting ligands to promote selective uptake by cancer cells. Carbohydrate-based targeting strategies are particularly attractive because tumor cells frequently exhibit altered glucose metabolism and overexpression of glycan-binding receptors, which can be explored to enhance PS accumulation. Chlorin e6 (Ce6), a second-generation PS characterized by strong absorption in the red region (~654 nm) and high ^1^O_2_ generation efficiency, was incorporated into a galactose-functionalized AuNP platform designed for targeted PDT. Following an initial screening of different carbohydrate ligands, galactose was selected because it is preferentially taken up by BC cells compared with non-tumorigenic mammary cells (MCF-10A). The resulting glyconanosystem consisted of 16 nm AuNPs conjugated to galactosyltriazole-PEG3-SH and Ce6. After 3 h of incubation, SKBR-3 (a HER2-positive BC model) and MDA-MB-231 cells were irradiated at 633 nm (29 mW/cm^2^ for 6 min). The galactose-targeted nanosystem significantly reduced SKBR-3 cell viability by up to 48%, whereas no photodynamic effect was observed in MDA-MB-231 cells. Further investigations suggested that the lack of efficacy in MDA-MB-231 cells was associated with the conjugate’s intracellular localization within acidic organelles, potentially promoting Ce6 degradation and impairing its photodynamic activity. These findings highlight both the potential and the cell-type dependence of glycan-mediated targeting strategies for AuNP-assisted PDT (Thomas-Moore et al. 2023).

Besides carbohydrate-mediated targeting, other biomolecule-based strategies have been explored to enhance the selective accumulation of PSs in tumor cells. Biotin is promising for this purpose because it participates in essential metabolic pathways and is often overexpressed in rapidly proliferating cancer cells. Building on this concept, two asymmetric porphyrins [5, 10,15-tris (4-nitrophenyl)−20-hydroxylphenylporphyrin], one of them containing a gallium center, were conjugated to biotinylated Au–Ag bimetallic NPs composed of an Au core coated with Ag and PEG. The asymmetric porphyrin design was intended to enhance triplet-state formation and consequently ^1^O_2_ generation, while the Au–Ag nanoplatform improved PS solubility and delivery. DLS (dynamic light scattering) measurements revealed particle sizes ranging from 7.5 to 9.9 nm after porphyrin loading. After 24 h of incubation, MDA-MB-231 cells were irradiated with a 625-nm LED for 30 min. All formulations showed negligible dark toxicity, whereas PDT markedly reduced cell viability. The gallium-containing conjugate (2-B-Au–Ag) displayed the highest photodynamic activity, reducing cell viability to 24.7%, while the non-metalated conjugate (1-B-Au–Ag) and PSs alone were less effective. Interestingly, the biotinylated Au–Ag NPs alone also reduced cell viability to approximately 32%, suggesting an intrinsic contribution of the nanoplatform to the overall phototoxic effect (Magaela et al. 2025).

Further refinements in AuNP-based PDT platforms have combined PSs derived from the metalation of free-base tetrakis-(4-methylthiophenyl)porphyrin with carbohydrate functionalization to improve both photophysical properties and cellular uptake. In this approach, zinc-, gallium-, and indium-containing porphyrins were conjugated to citrate-stabilized AuNPs via thiol groups, thereby generating nanosystems designed to enhance PS delivery. In addition, sugar-functionalized gallium and indium porphyrins (GaPS and InPS) were prepared to promote cellular internalization. TEM analysis revealed AuNPs with an average diameter of 2.5 nm. Following incubation with MDA-MB-231 cells, PDT was performed using a 415 nm LED for 30 min. All AuNP conjugates exhibited enhanced photodynamic activity compared with the corresponding free porphyrins, confirming the beneficial role of the metallic nanocarrier. Among the formulations, AuNPs-InPS exhibited the highest phototoxicity, reducing cell viability to approximately 20%, a result attributed to its superior ^1^O_2_ quantum yield. Furthermore, the incorporation of sugar moieties increased cellular uptake and further improved PDT efficacy. These findings highlight the synergistic contribution of AuNP-mediated delivery, porphyrin metalation, and carbohydrate functionalization (Vukeya et al. 2025).

Beyond their role as delivery platforms, metallic NPs can contribute to PDT through plasmonic effects that can enhance ROS generation. Temoporfin (mTHPC), a second-generation chlorin PS known for its clinical use in PDT, was incorporated together with AuNPs into a star-like polymer nanocarrier composed of a dextran core and anionic polyacrylamide arms with a plasmon band at 520 nm. To evaluate the contribution of the metallic component, a control formulation lacking AuNPs was also prepared. DLS revealed a hydrodynamic diameter of approximately 90 nm for the nanocomplex, while ROS measurements showed substantially greater ROS generation in the AuNP-containing formulation than with mTHPC alone following irradiation, although the excitation light did not fully overlap with the plasmon band. MDA-MB-231 cells were irradiated at 660 nm (93.6 mW/cm^2^ for 10 min), resulting in an IC50 of 4 µg/mL for the AuNP-loaded carrier containing 0.4 µg/mL mTHPC, which outperformed PS alone. Additionally, cancer cells showed morphological alterations consistent with apoptosis, whereas no noteworthy effects were detected in non-tumorigenic MCF-10A cells (Yeshchenko et al. 2024).

Anisotropic nanostructures have also attracted interest because their optical properties can be designed through shape engineering. In this context, Au nanobipyramids (AuBPs) were combined with a thioacetate-modified dimeric phthalocyanine PS to generate a pH-responsive nanosystem that releases the PS under acidic conditions. The AuBPs measured approximately 45 × 80 nm and exhibited a plasmon resonance centered at 785 nm, whereas the phthalocyanine absorbed around 610 nm. Following 12 h of incubation, MCF-7 cells were irradiated at 610 nm (40 mW/cm^2^ for 20 min). The nanosystem exhibited negligible dark toxicity but pronounced phototoxicity upon irradiation, resulting in nearly complete loss of cell viability at 10 nM and an IC50 of approximately 1.7 nM (Cao et al. 2024).

Similar liposome-based delivery strategies have also been investigated using AgNPs. Zinc phthalocyanine tetrasulfonate (ZnPcS_4_) is a phthalocyanine PS characterized by strong absorption in the red region (~670 nm). AgNPs synthesized via a green approach using *Dicoma anomala* were co-encapsulated with ZnPcS_4_ in liposomes to enhance PS delivery and therapeutic efficacy. The AgNPs exhibited an average diameter of 36 nm. PDT was evaluated in MCF-7 cells and hTERT-HME1 mammary epithelial cells using 660 nm irradiation. All formulations showed negligible dark toxicity and induced dose-dependent phototoxicity upon irradiation. Among the tested systems, the AgNPs-Lip@ZnPcS_4_ nanocomplex displayed the highest efficacy, with the lowest IC50 (3.7 µg/mL), and outperformed formulations containing either AgNPs or ZnPcS4 alone. The nanosystem also promoted ^1^O_2_ generation, preferential intracellular accumulation, mitochondrial damage, and activation of apoptotic pathways. These findings demonstrate that liposomal co-delivery of AgNPs and ZnPcS_4_ can enhance photodynamic responses against BC cells (Chota et al. 2025).

An alternative strategy involves using the PS itself as a stabilizing and functional coating for metallic NPs. Sodium copper chlorophyllin (CHL), a chlorophyll-derived third-generation PS with absorption in the red region, was employed both as a PS and as a surface-coating agent for AgNPs synthesized by chemical reduction. The resulting AgNPs@CHL exhibited a spherical morphology with an average diameter of approximately 25 nm (TEM). PDT efficacy was evaluated in MDA-MB-231 cells using 650 nm LED irradiation (50 mW/cm^2^ for 15 min). While cell viability remained above 80% in the absence of light, irradiation induced a marked cytotoxic effect, with an IC50 of 6.25 µg/mL and cell viability below 10% at the highest concentration tested. Microscopy studies demonstrated efficient cellular internalization and predominant cytoplasmic localization of the nanocomplex. Furthermore, irradiation triggered a pronounced apoptotic response, with approximately 60% of cells undergoing early apoptosis compared with only 15% in non-irradiated controls (Abdel Gaber et al. 2025).

Overall, associating PSs with metal-based nanocarriers can improve aqueous dispersibility and delivery, reduce PS dark toxicity, increase cellular uptake (especially via functionalization), and ultimately enhance photodynamic efficacy against BC cells. Beyond serving as passive carriers, these nanoplatforms can actively influence PDT outcomes by modulating intracellular trafficking, subcellular localization, and ROS generation. Particularly, the studies suggest that therapeutic efficacy depends not only on increasing PS accumulation within tumor cells but also on controlling its intracellular fate and interactions with specific cellular pathways. In addition, only one study explored plasmonic effects, although this phenomenon appears highly promising for further enhancing PDT responses. The contrasting outcomes observed across different BC cell lines further highlight that NP performance is strongly influenced by tumor-specific biological characteristics, underscoring the importance of designing nanosystems tailored not only to the PS but also to the target tumor phenotype. Nevertheless, experimental parameters vary even more in PDT than in PTT, including differences in irradiation conditions, NP composition and architecture, PS type, and targeting strategies, which hinders direct comparisons between studies. As in PTT, most investigations remain restricted to in vitro models. Thus, future works should focus on standardization, mechanistic understanding, and preclinical validation to translate AuNP- and AgNP-assisted PDT from promising laboratory findings into clinically relevant therapies for BC.

## Au and Ag nanoparticles in radiotherapy

### Basic principles

**Fig. 3:**
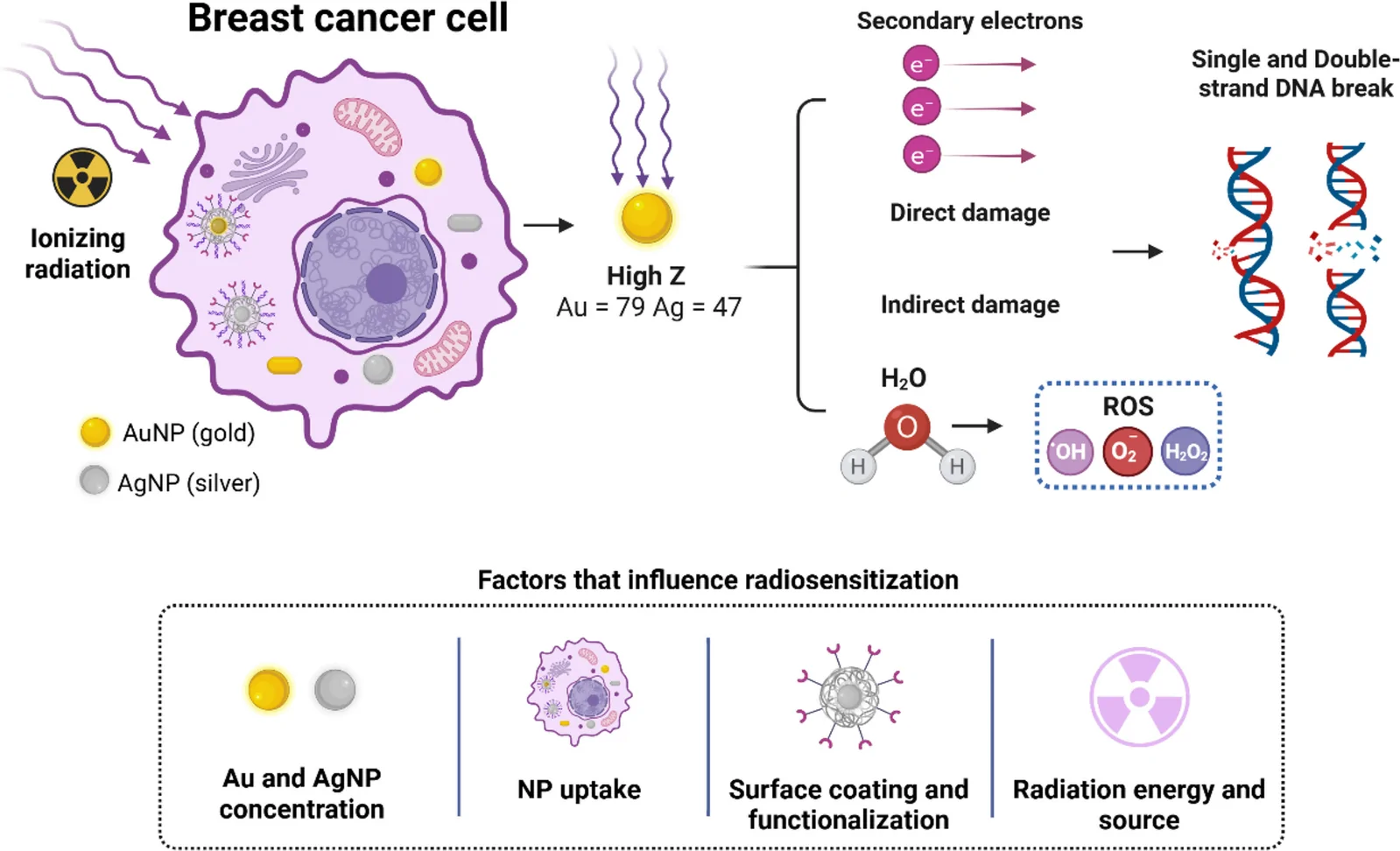
Schematic representation of the radiosensitization mechanisms mediated by metallic NPs in BC cells. Following exposure to ionizing radiation, high-atomic-number (high-Z) NPs, such as AuNPs and AgNPs, enhance local energy deposition through increased photon–matter interactions. This process generates secondary electrons that directly damage cellular biomolecules, particularly DNA, resulting in single- and double-strand breaks. Simultaneously, radiation-induced water radiolysis generates ROS, which contributes to indirect oxidative damage. Several factors influence the magnitude of radiosensitization, including NP concentration, cellular internalization and intracellular localization, surface coating and functionalization, and radiation source and energy. Together, these parameters determine the extent of radiation dose amplification and the resulting biological response. Created with BioRender.com, agreement number EP29SD2DXI

AuNPs and AgNPs have gained prominence for enhancing the therapeutic efficacy of RT in BC, primarily because they can selectively increase radiation-induced damage in tumor tissues. The rationale for using these metallic nanostructures as radiosensitizers lies in their ability to couple nanoscale physical dose amplification with enhanced chemical and biological damage, ultimately improving tumor control without increasing systemic toxicity (Fig. 3).

Physically, this radiosensitizing capability is linked to the high atomic numbers of Au (*Z* = 79) and Ag (*Z* = 47), which significantly increase the probability of photoelectric interactions with ionizing radiation compared to soft tissue. As a result, AuNPs and AgNPs act as local amplifiers of radiation dose at the nanoscale, providing a strong basis for their therapeutic application (Jackson et al. 2024). When exposed to ionizing radiation, these nanomaterials absorb energy and emit secondary electrons, including photoelectrons, Compton recoil electrons, and Auger electrons. These electrons travel only short distances, leading to highly localized ionization events near the NPs. This means that while the overall radiation dose to the patient remains unchanged, damage to important cellular components of cancer cells, such as DNA, becomes much more pronounced. This precise energy deposition is crucial for enhancing the effectiveness of RT in BC (Her et al. 2017).

Beyond physical effects, AuNPs and AgNPs can contribute to radiosensitization through chemical processes. The secondary electrons generated by these nanostructures interact with water molecules, producing ROS. This increased oxidative stress can damage vital cellular components, intensifying therapeutic impact. Many BC cells already have a disrupted redox balance, so this additional oxidative burden can push them beyond their limits, thereby making treatment more successful.

From a therapeutic perspective, these radiosensitizing properties can be explored through different strategies in BC. Systemic administration allows passive accumulation in tumors via the EPR effect, while surface functionalization enables active targeting of tumor-associated biomarkers, improving intratumoral retention and cellular uptake. In addition, nanobrachytherapy (NBT) represents a promising approach in which radioactive NPs, such as ^198^AuNPs, are delivered directly into the tumor, enabling highly localized radiation dose deposition while minimizing exposure to the surrounding healthy tissues (Ghosh et al. 2024). In this setting, the physical and chemical amplification effects are maximized locally, enabling highly efficient dose enhancement where it is needed. This approach can be especially attractive for BC requiring high local control with minimal damage to surrounding healthy tissue.

Hence, the unique physical, chemical, and biological properties of AuNPs and AgNPs provide a strong rationale for their use as radiosensitizers in BC. The following studies report how these mechanisms have been explored to improve radiation responses through different NP designs and delivery strategies.

### Applications in radiotherapy

Recent studies employing relatively simple AuNP formulations have provided insights into the mechanisms underlying radiosensitization under clinically relevant megavoltage irradiation (Table 3). Using green-synthesized AuNPs coated with polyphenols from *Laurus nobilis* leaf extract and displaying mixed spherical (~40 nm) and triangular (~180 nm) morphologies, irradiation of MCF-7 cells with 2.67 and 8 Gy resulted in enhanced radiotoxicity, with AuNPs (0.46 µg/mL) reducing cell viability by up to 50% and promoting extensive DNA fragmentation, F-actin disorganization, and nuclear (Tarantino et al. 2025).

**Table 3:** Summary of AuNP- and AgNP-mediated RT in BC models

NP type	Size (nm)	Coating/Functionalization	Experimental Model	Irradiation source and parameters	NP Concentration	Main Outcomes	Ref
Au nanospheres	16–18	PEG + transferrin	In vitro 4T1	X-rays 160 kV 1.93 Gy	1 nM	AuNPs did not influence initial DSBs but delayed DSB repair	Atkinson et al. (2025)
Au nanospheres	5 and 10 (TEM)	-	In vitro MCF-7	X-rays Linac (6 MV) 1, 2, and 4 Gy	50 µg/mL (after 2,5, 5, 10, and 50 µg/mL for MTT)	Cellular kinetics decreased in all experimental conditions, with 50 µg/mL AuNPs reducing proliferation	Engelbrecht-Roberts et al. (2025)
Ag nanospheres	< 50 (TEM)	Anti-HER2 antibody (herceptin) via pMBA linker	In vitro SKBR3	Gamma (^60^Co) 0,1, 2, 4, and 6 Gy	7 µg/mL	Enhanced radiosensitivity; molecular changes detected via Raman correlated with resistance/dose	Jafarzadeh et al. (2024)
Au nanospheres, nanorods, and nanocages	25 (spheres) 35 (rods) 45 (cages) (TEM)	-	In vitro 4T1 in vivo 4T1 tumor-bearing BALB/c mice	X-rays 320 keV 2, 4, and 8 Gy (in vitro) 12 Gy (in vivo)	In vitro: varied according to the surface density In vivo: 50 µg of AuNPs (1 mg/mL)	Au nanocages exhibited the highest per-particle radiosensitization, followed by spheres and rods. Greater tumor growth delay in vivo	Jenkins et al. (2023)
Ag nanospheres	81	-	In vitro MDA-MB-231 and MCF-7	X-ray 6 MV 4 and 8 Gy	5 µg/mL (for combination studies)	Synergistic effect (CI = 0.49); increased apoptosis and ROS; decreased HIF-1α, cyclin D1, and p-ERK	Montazersaheb et al. (2024)
Ag nanospheres	50–80 (SEM)	PVP	In vitro MDA-MB-231 and MDA-MB-468	X-ray 6 MV 4 and 8 Gy	2 and 4 µg/mL (MDA-MB-231) 5 and 7 µg/mL (MDA-MB-468)	CI < 1; inhibition of migration and angiogenesis; reduction of VEGFR2, Tie2, MMP2, and MMP9	Montazersaheb et al. (2026)
Au nanospheres	10.5 ± 1.3 and 30.9 ± 1.8 (TEM)	PEG (800 and 2000) − molecular weight + RGD peptide	In vitro MCF-7 (2D and 3D models)	Gamma (^137^Cs) 2 Gy	0.4–20 µg/mL	Significant differences in uptake and ROS generation between 2 and 3D models. Shorter PEG chains were more effective	Musielak et al. (2023a)
Au nanospheres	10.5 ± 1.3 and 30.9 ± 1.8 (TEM)	PEG (800 and 2000) − molecular weight + RGD peptide	In vitro MDA-MB-231	Gamma (^137^Cs) 2 Gy	0.4–20 µg/mL	Radiosensitization depends on incubation time. SF decreased after 30 min pre-incubation, but increased after 3 and 24 h	Musielak et al. (2023b)
^198^Au nanospheres	~5 (TEM) ~50 (DLS)	Gum arabic	In vivo 4T1 tumor-bearing BALB/c mice	Intratumoral inoculation, ~200 µCi; estimated tumor dose rate ~9.34 mGy/s, decreasing to ~4.14 mGy/s after 20 min	~1.4 × 10^14^ NPs/injection	Reduced tumor growth, pulmonary metastases, splenomegaly, and Gal-3 expression; preferential tumor retention	Pinto et al. (2026)
Au nanospheres	18 (TEM) 21.4 (DLS)	Alginate + triptorelin peptide	In vitro MCF-7	X-rays Linac (6 MeV) 2, 4, 6, and 8 Gy	20 µg/mL	DEF increased up to 2.32 at 4 Gy for AuNPs compared to radiation alone	Tavakkoli et al. (2025)
Au nanospheres	10.3 ± 3.7 (TEM)	Chitosan	In vitro 4T1	X-rays Linac 6 MV 4 Gy	30, 60, 120, and 240 µg/mL	Cell viability reduced to < 60% with radiation vs ~80% without radiation	Wan et al. (2024)

*CI* combination index, *DEF* dose enhancement factor, *DSBs* DNA double-strand breaks, *HER2* human epidermal growth factor receptor 2, *HIF-1α* hypoxia-inducible factor 1 alpha, *IC50* half-maximal inhibitory concentration, *Gal* galectin, *Linac* linear accelerator, *MMP* matrix metalloproteinase, *p-ERK* phosphorylated extracellular signal-regulated kinase, *PEG* polyethylene glycol, *pMBA* para-mercaptobenzoic acid, *PVP* polyvinylpyrrolidone, *RGD* arginine-glycine-aspartic acid peptide, *ROS* reactive oxygen species, *SEM* scanning electron microscopy, *TEM* transmission electron microscopy, *Tie2* tyrosine kinase with immunoglobulin-like and EGF-like domains 2, *VEGFR2* vascular endothelial growth factor receptor 2

In a complementary mechanistic study, citrate-coated AuNPs with diameters of 5 and 10 nm, as determined by TEM, increased G2/M arrest and micronucleus formation in irradiated MCF-7 cells following 2 Gy exposure, indicating enhanced chromosomal instability and cell cycle-associated radiosensitivity, although direct DNA damage was limited by the NPs’ predominantly vesicular localization (Engelbrecht-Roberts et al. 2025). Similarly, chitosan-coated AuNPs with a diameter of ~10 nm (as determined by scanning TEM) enhanced the effects of 4 Gy irradiation in murine 4T1 TNBC cells, primarily by increasing intracellular ROS production (Wan et al. 2024).

Beyond passive cellular uptake, the radiosensitizing performance of AuNPs is critically influenced by the intracellular localization of Au at the moment of irradiation, since short-range secondary electrons and locally generated ROS produced by interactions of photons with electrons act mainly on targets in their immediate vicinity. Surface functionalization with peptides, proteins, antibodies, aptamers, or carbohydrate-based ligands has therefore been pursued to drive AuNPs into receptor-mediated internalization pathways, thereby raising a local Au concentration in tumor cells while improving colloidal stability and reducing non-specific toxicity (Sanità et al. 2020).

Alginate-coated AuNPs functionalized with triptorelin, a gonadotropin-releasing hormone (GnRH) analog, significantly enhanced radiosensitivity in MCF-7 cells exposed to 6 MV X-rays, producing a dose enhancement factor (DEF) of 2.32 at 4 Gy together with increased ROS generation and apoptosis (Tavakkoli et al. 2025). Similarly, glucose- and mannose-functionalized ultrasmall AuNPs embedded in a cellulose matrix improved uptake by MDA-MB-231 cells and achieved a DEF of 1.82 under 250 kVp irradiation, accompanied by increased DNA fragmentation and γH2AX expression, a marker of DNA double-strand break (DSB) responses. However, enhanced uptake does not necessarily translate into increased primary DNA damage (Biagiotti et al. 2024). In 4T1 TNBC cells, transferrin-functionalized PEGylated AuNPs did not increase early DSB formation following 160 kV irradiation, as assessed by the STRIDE (sensitive recognition of individual DNA ends) assay, despite evidence of persistent DNA damage signaling and delayed γH2AX activation. These findings suggest that radiosensitization may also arise from impaired DNA repair processes rather than solely from enhanced induction of DNA lesions (Atkinson et al. 2025). Further evidence of the importance of biological context was provided by studies employing arginine–glycine–aspartic acid (RGD)-PEG-functionalized AuNPs, which target integrins overexpressed in tumor cells and tumor-associated vasculature. While 30 nm RGD-PEG2000-AuNPs reduced clonogenic survival and increased DNA damage in two-dimensional MCF-7 cultures following 2 Gy irradiation from a ^137^Cs source, these effects were largely absent in three-dimensional models, where limited NP penetration and greater radioresistance reduced treatment efficacy (Musielak et al. 2023a). Additional investigations demonstrated that radiosensitization depended strongly on NP size, PEG chain length, concentration, incubation time, and cell type, with the most pronounced effects observed for 30 nm RGD-functionalized AuNPs following short pre-irradiation incubation periods (Musielak et al. 2023b).

Beyond surface functionalization, NP morphology has emerged as another key parameter influencing radiosensitization. Shape variations can alter energy deposition patterns, ROS generation, cellular interactions, and ultimately the biological response to ionizing radiation. To investigate these effects, Au nanocages (~45 nm), nanospheres (~25 nm), and nanorods (~15 × 35 nm) were compared in BC models. Radiosensitization was strongly morphology-dependent, with nanocages producing the greatest enhancement, followed by nanospheres and nanorods. Even at low NP densities, AuNP exposure significantly increased radiation-induced oxidative stress, DNA DSBs, and protein oxidation, indicating efficient amplification of radiation damage. Importantly, these effects translated in vivo, where intratumoral administration of Au nanocages combined with a single 12 Gy dose significantly delayed the growth of 4T1 TNBC tumors, increasing the time required for tumor volume to quadruple from 13 to 18 days. These findings demonstrate that NP geometry represents an important design parameter for optimizing radiation dose amplification while potentially reducing the amount of Au required to achieve a therapeutic benefit (Jenkins et al. 2023).

While conventional approaches rely on AuNPs to enhance the effects of externally delivered radiation, radioactive AuNPs offer an alternative strategy in which the radiation source is generated directly within the NP. In a recent in vivo study, ^198^AuNPs synthesized from neutron-activated ^197^Au were administered intratumorally at an activity of approximately 200 µCi in a syngeneic 4T1 mouse model. NBT significantly reduced tumor growth, pulmonary metastatic burden, splenomegaly, and galectin-3 expression compared with untreated controls. Biodistribution analysis demonstrated preferential tumor retention of the radioactive NPs, while Monte Carlo simulations estimated a tumor dose rate of approximately 9.34 mGy/s immediately after administration, decreasing to 4.14 mGy/s after 20 min as the NPs diffused within and beyond the tumor. The simulations also characterized the spatial distribution of radiation emitted by the intratumorally administered ^198^AuNPs and estimated the dose delivered to surrounding tissues and the environment. This dosimetric assessment highlights an important translational aspect of radioactive NP-based RT, in which radiation protection and environmental exposure must be considered alongside therapeutic efficacy (Pinto et al. 2026).

Similar to AuNPs, conventional AgNPs have demonstrated intrinsic radiosensitizing properties in BC models, largely attributed to their ability to amplify oxidative stress and enhance radiation-induced cell death (Table 3). In TNBC cell lines (MDA-MB-231 and MDA-MB-468), combined treatment with AgNPs and ionizing radiation markedly reduced cell viability, with survival rates decreasing to approximately 23–29%. This effect was accompanied by substantial increases in intracellular ROS production and apoptosis, highlighting oxidative stress as a major contributor to radiosensitization. Beyond enhancing radiation-induced cytotoxicity, AgNPs also suppressed the migratory and angiogenic behaviors of key regulators of angiogenesis and invasion, including downregulating vascular endothelial growth factor receptor 2 (VEGFR2), tyrosine kinase with immunoglobulin-like and EGF-like domains 2 (Tie2), matrix metalloproteinase-2 (MMP2), and MMP9 (Montazersaheb et al. 2026).

To improve tumor selectivity, AgNPs have also been functionalized with ligands targeting molecular markers associated with specific BC subtypes. In this context, HER2-conjugated AgNPs were developed for HER2-overexpressing SKBR3 cells. Successful conjugation was confirmed by shifts in the plasmon spectrum, while biological assays demonstrated radiation-dose-dependent reductions in cell viability. In addition, Raman spectroscopic analyses revealed treatment-induced molecular alterations consistent with enhanced radiation responses (Jafarzadeh et al. 2024).

Green synthesis strategies have been used as alternatives for producing AgNPs with improved sustainability and biological compatibility while preserving radiosensitizing activity. Using pumpkin peel extract as a reducing and stabilizing agent, stable spherical AgNPs were produced and evaluated in BC models exposed to radiation. Combined treatment significantly decreased cell viability and increased apoptosis compared with either modality alone. Mechanistic analyses revealed reduced expression of hypoxia-inducible factor 1 alpha (HIF-1α), an increased Bax/Bcl-2 ratio, and upregulation of p53, indicating suppression of hypoxia-associated survival pathways and activation of mitochondrial apoptosis. Interestingly, the radiosensitizing effect was more pronounced in TNBC cells than in hormone receptor-positive MCF-7 cells, suggesting that a tumor subtype may influence the therapeutic benefits of AgNP-mediated radiation enhancement (Montazersaheb et al. 2024).

Collectively, these studies demonstrate the potential of AuNPs and AgNPs as radiosensitizers in BC, highlighting their ability to amplify radiation-induced oxidative stress, enhance DNA damage signaling, promote apoptosis, and suppress tumor-associated processes, including migration and angiogenesis. The findings indicate that radiosensitization is governed not only by the physical properties of high-Z nanomaterials but also by complex biological factors, including intracellular localization, cellular uptake pathways, DNA repair dynamics, and tumor-specific characteristics. Surface functionalization with targeting ligands has emerged as a promising strategy to improve tumor selectivity and intracellular accumulation. However, enhanced uptake does not necessarily translate into proportional increases in primary DNA damage, suggesting that mechanisms such as persistent oxidative stress and impaired DNA repair may play equally important roles in determining therapeutic outcomes. Likewise, the superior performance observed for specific NP geometries highlights that morphology can substantially influence energy deposition and biological responses to irradiation. Nevertheless, substantial heterogeneity in NP composition, size, shape, surface chemistry, irradiation parameters, and biological models continues to hinder direct comparisons between studies. Therefore, despite the encouraging preclinical evidence, further mechanistic investigations, protocol standardization, and in vivo studies will be important to establish robust design principles and accelerate the clinical translation of NP-assisted RT for BC.

## Applications of AuNPs in bimodal photon therapies

Unlike the individual therapeutic modalities discussed above, all studies identified as bimodal approaches were based exclusively on AuNP-containing nanosystems (Table 4). This trend may be attributed to the unique physicochemical and optical properties of AuNPs, which make them particularly well suited for developing multifunctional therapeutic platforms. Accordingly, integrating photodynamic, photothermal, and radiation-induced mechanisms within a single AuNP-based nanoplatform has emerged as a promising strategy to overcome the limitations of monotherapies and enhance antitumor efficacy in BC, as presented herein.

**Table 4:** Summary of AuNP-based bimodal photon therapies in BC models

NP type	Size (nm)	Coating/Functionalization	PS/precursor type	Experimental model	Radiation |Energy| Dose (Gy)	Light parameters	NP/PS concentration	Main outcomes	Ref
***PDT* + *PTT***									
Au nanospheres	29.3 (TEM)	HTCC	Methylene blue	In vitro MDA-MB-231	N/A	660-nm laser, 100 mW/cm^2^ Warm white LED, 55.3 J/cm^2^, 1800s	AuNP: 1.35 × 10^−10^ to 6.75 × 10^−10^ M MB: 0.5, 1.0, and 2.5 µM/L	HTCC-MB-AuNPs convert 25% of absorbed photon energy into local heat and generate dual ROS (^1^O_2_ and ^**·**^OH for PDT)	Sangaletti et al. (2024)
Au nanorods	16 × 52 (TEM) TPA-BPAF-SiPc@AuNR@SiO_2_: 39 × 75 (TEM)	Mesoporous silica	Phthalocyanine	In vitro MCF-7	N/A	PDT: 671 nm, 100 mW/cm^2^, 300 s PTT: 808 nm, 300 mW/cm^2^, 300 s	Au (100 µg/mL using ICP-OES) Cytotoxic assay (0–100 µg/mL)	PDT + PTT reduced cell viability to 13.1% (vs 50.6% PDT alone, 33.5% PTT alone)	Shen et al. (2025)
***RT* + *PDT***									
Au nanospheres	15 (TEM)	PEG	Porphyrin	In vitro MDA-MB-231 (2D and 3D models)	X-rays, 225 kVp, 2 Gy	660-nm laser, 100 mW, 40 J/cm^2^, 40 s	35 µg/mL (Au) + 25 µM (TMPyP)	Survival fraction reduced to ~40% at 2 Gy vs. ~80% in control; CDI < 1, indicating synergy	Silva et al. (2026)
***RT* + *PTT***									
Au nanorods	NI	Chitosan (delivered inside cellular vehicles)	N/A	In vitro MCF-7 and MDA-MB-231	X-rays, 6 MV, 2 Gy	808-nm laser, 1 W/cm^2^, 300 s	100 µM Au	Hyperthermia plus radiotherapy reduced colony formation and increased DNA damage compared to treatment alone	Anceschi et al. (2025)
Au nanospheres	8.7 ± 1.3 (TEM)	alginate-dopamine	N/A	In vitro 4T1	X-rays, 6 MV, 4 Gy	808-nm laser, 1 W/cm^2^, 300 s	500 µg/mL	Cell viability reduced to ~35% with NPs + X-ray + NIR vs ~84% (X-ray alone) and ~95% (NIR alone)	Ghaffarlou et al. (2024)

*BPAF* bisphenol AF, *CDI* coefficient of drug interaction, *HTCC N*-[(2-hydroxy-3-trimethylammonium)propyl] chitosan chloride, *ICP-OES* inductively coupled plasma optical emission spectrometer, *MB* methylene blue, *NIR* near-infrared, *PEG* polyethylene glycol, *ROS* reactive oxygen species, *SiO*_*2*_ silicon dioxide, *SiPc* silicon phthalocyanine, *TEM* transmission electron microscopy, *TPA* triphenylamine, *TMPyP* 5,10,15,20-tetrakis(1-methyl-4-pyridinio)porphyrin, *N/A* not applicable, *NI* not informed

Among the strategies explored for bimodal phototherapy, integrating PDT and PTT into a single nanosystem has attracted considerable attention because the simultaneous generation of ROS and localized hyperthermia can produce complementary cytotoxic effects. In this context, mesoporous silica-coated Au nanorods (AuNR@SiO_2_) conjugated with a fluorinated triphenylamine-substituted silicon phthalocyanine (TPA-BPAF-SiPc) were engineered for mitochondrial-targeted PDT/PTT in MCF-7 cells. Structurally, TEM analysis demonstrated that bare AuNRs presented dimensions of approximately 52 × 16 nm, which increased following silica coating and phthalocyanine conjugation. The AuNRs exhibited a transverse plasmon resonance peak at 514 nm and a longitudinal plasmon resonance peak at 814 nm. Silica coating induced a slight redshift of the longitudinal band, while subsequent phthalocyanine conjugation was confirmed by the appearance of its characteristic absorption peak at 693 nm in the resulting nanoconjugate. The nanosystem promoted efficient cellular internalization, preferential mitochondrial localization, and lysosomal escape, thereby improving intracellular therapeutic delivery. Upon NIR irradiation at 808 nm (0.5 W/cm^2^), the NPs exhibited excellent photothermal performance, raising the temperature to 67.5 °C within 15 min. In parallel, strong intracellular ROS generation was observed following 671-nm laser irradiation (100 mW/cm^2^, 5 min), confirming the platform’s photodynamic activity. While PDT and PTT alone reduced cell viability to 50.6% and 33.5%, respectively, the combined treatment decreased viability to 13.1%, demonstrating a pronounced synergistic effect. Apoptosis and necrosis analyses further confirmed that the simultaneous induction of oxidative and thermal damage substantially enhanced cancer cell killing (Shen et al. 2025).

A simpler approach to PDT/PTT integration employed methylene blue (MB)-loaded AuNPs stabilized with *N*-(2-hydroxy)propyl-3-trimethylammonium chitosan chloride (HTCC) in MDA-MB-231 cells. In this nanosystem, HTCC improved the stability and biocompatibility of the NPs, while AuNPs (29.3 nm, TEM) served as both nanocarriers and photothermal mediators. UV–Vis spectroscopy confirmed functionalization, and AuNPs exhibited a plasmon peak at 530 nm. Thermal lens analysis revealed that approximately 25% of the absorbed light energy was converted into heat, and ROS assays confirmed the generation of ^1^O_2_ and other ROS, albeit at lower levels than with free MB, supporting the simultaneous activation of photothermal and photodynamic mechanisms. The formulation exhibited minimal dark toxicity, maintaining approximately 85% cell viability even at the highest MB concentration tested (2.5 µM). In contrast, irradiation with a warm white LED (55.3 J/cm^2^, 30 min) reduced cell viability to nearly 10%, even at the lowest MB concentration evaluated, a level similar to that observed with free MB (Sangaletti et al. 2024).

The integration of RT and PDT has also been explored by directly incorporating PSs onto AuNP surfaces, demonstrating promising efficacy in RT/PDT applications. Porphyrin-coated AuNPs (AuNPs@TMPyP) were evaluated in MDA-MB-231 TNBC cells and MCF-10A cells cultured as both monolayers and spheroids. AuNPs with average core diameters of approximately 15 nm were synthesized, followed by PEG functionalization and coating with the cationic porphyrin TMPyP, resulting in hydrodynamic diameters near 30 nm. Following PDT activation with a 660-nm laser (100 mW for 40 s) and subsequent irradiation with 225 kVp X-rays (2 Gy), the nanosystem induced pronounced oxidative stress by reducing thioredoxin reductase (TrxR) activity while increasing ROS and ^1^O_2_ production. These effects triggered mitochondrial depolarization, apoptosis, and necrotic cell death, ultimately leading to a substantial reduction in clonogenic survival. Importantly, the combination of PDT and RT produced synergistic cytotoxic effects, as indicated by combination drug interaction values below 1. Furthermore, the preferential accumulation of AuNPs@TMPyP in tumor cells compared with healthy epithelial cells highlighted the potential of this platform to enhance therapeutic selectivity (Silva et al. 2026).

The combination of RT and PTT has also emerged as an attractive strategy for enhancing radiation responses while simultaneously inducing localized thermal damage. By increasing tumor temperature, photothermal agents can impair DNA repair pathways, disrupt protein homeostasis, and improve radiation sensitivity, thereby complementing the cytotoxic effects of ionizing radiation. In this context, alginate–dopamine-functionalized AuNPs were synthesized and evaluated as photothermal radiosensitizers in the 4T1 TNBC model. The Au@Alg-DA NPs exhibited an average size of 8.7 nm, a hydrodynamic diameter of around 95 nm, and a plasmon peak at 540 nm. Upon exposure to 6 MV X-rays (4 or 8 Gy) and NIR irradiation (808 nm, 1 W/cm^2^), the nanosystem exhibited efficient photothermal conversion, raising the temperature to approximately 45 °C while maintaining excellent biocompatibility. Notably, the combination of AuNPs, X-ray irradiation, and NIR activation produced the strongest antitumor response, reducing cell viability to approximately 35% and inducing extensive tumor necrosis without detectable systemic toxicity (Ghaffarlou et al. 2024).

More sophisticated RT/PTT platforms have incorporated biological carriers to improve NP delivery and tumor targeting. In this regard, endothelial colony-forming cells (ECFCs) loaded with chitosan-coated AuNRs were employed as cellular vehicles capable of homing to hypoxic tumor regions. The AuNRs displayed a strong plasmon peak in the NIR region (780–810 nm) and were activated using an 808-nm laser (1.0 W/cm^2^, reaching 43 °C), while RT was delivered at 2 Gy. ECFCs internalized up to tenfold more AuNRs than BC cells (MCF-7 and MDA-MB231 lineages), enabling efficient NP transport without significant toxicity. Combined RT/PTT treatment significantly increased DNA damage, as evidenced by COMET assays and γH2AX expression, and markedly reduced clonogenic survival compared with monotherapies (Anceschi et al. 2025).

Together, these studies demonstrate the potential of AuNP-based bimodal platforms to improve therapeutic efficacy against BC by integrating complementary mechanisms of action within a single nanosystem. The simultaneous generation of ROS and hyperthermia in PDT/PTT, the combination of photodynamic and radiation-induced oxidative damage in RT/PDT, and the integration of thermal stress with radiosensitization in RT/PTT yield greater antitumor effects than the respective monotherapies. The findings indicate that the advantages of bimodal therapy are not merely additive but result from complex interactions that intensify cellular stress, disrupt multiple survival pathways, and enhance susceptibility to treatment-induced cell death. These studies further underscore the versatility of AuNPs as multifunctional platforms that can carry PSs and targeting ligands, be encapsulated in biological vehicles, and facilitate photothermal conversion and radiation enhancement. However, the current body of research is limited, with most studies conducted in vitro. Bimodal approaches inherit many of the challenges associated with individual PTT, PDT, and RT strategies, since significant variability in NP design, irradiation protocols, treatment sequences, and biological models also hinders direct comparisons and the identification of optimal therapeutic strategies. Consequently, while existing evidence supports the potential of AuNP-mediated bimodal therapies, further mechanistic studies and comprehensive in vivo investigations are necessary to determine whether the observed combined effects translate into clinically meaningful outcomes for BC treatment.

## Future directions

Emerging strategies in Au and AgNP-mediated photon-based therapies for BC increasingly focus on developing more integrated nanoplatforms that enhance local retention, multifunctionality, and therapeutic integration. Rather than focusing only on the metallic core, emerging approaches aim to engineer the surrounding architecture of the NP system, including its matrix, surface, asymmetry, and combination with other therapeutic or diagnostic components.

Matrix-based systems may offer an option to improve local retention and spatial distributions of NPs. By incorporating NPs into structured biomaterials, these platforms may help maintain the therapeutic agent near the tumor site or surgical bed while reducing systemic dispersion and supporting localized light- or radiation-based activation. For example, cellulose nanocrystal-based systems have been proposed as Trojan-horse carriers for ultrasmall Au radiosensitizers, enhancing NP accumulation and increasing radiation-induced DNA damage (Biagiotti et al. 2024). Similarly, nRGO@AuNP-loaded PCL (polycaprolactone) nanofibrous scaffolds have been explored for localized PTT (Jaswal et al. 2024), where the scaffold architecture supports the spatial organization of the plasmonic system and enables efficient BC tumor cell destruction under NIR irradiation.

Hydrogels have also been developed as matrix-based systems for direct tumor application, enabling localized retention of NPs and bioactive molecules while enhancing their therapeutic effects. One strategy involved an AuNP-loaded hydrogel composed of chitosan and amphiphilic micelles based on hyaluronic acid, oleic acid, cysteamine, and all-trans retinoic acid, designed to combine the therapeutic properties of retinoic acid with AuNP-mediated PTT (Wang et al. 2023). This localized approach aimed to improve tumor control while providing anti-recurrence and anti-metastatic effects. Beyond preoperative applications, AuNP-containing hydrogels have also been explored for postsurgical tumor management (Kim et al. 2025). A hydrogel incorporating AuNPs and aptamers within a polymeric phenylboronic acid/polyvinyl alcohol matrix was developed for implantation after tumor resection. In vitro and in vivo assays demonstrated that this multifunctional platform promoted photothermal heating, ROS scavenging, and VEGF sequestration, thereby modulating the tumor microenvironment and reducing tumor recurrence through a multifaceted therapeutic approach.

Hybrid nanoplatforms offer another strategy by combining NPs with additional components to improve properties such as local retention, colloidal stability, or therapeutic activation. A representative example is the ^161^ Tb-labeled AuNP system functionalized with TADOTAGA, a DOTA-derived macrocyclic chelator that enables stable radionuclide coordination and immobilized onto maghemite iron oxide nanoflowers. This hybrid platform improved intratumoral retention compared with freely administered radiolabeled AuNPs and supported localized nanoscale brachytherapy (Salvanou et al. 2025). Other hybrid systems, such as Bi_2_S_3_-AuNRs, combine high-Z components for radiosensitization with NIR-responsive AuNRs for PTT (Saghatchi et al. 2025).

Janus NPs, anisotropic nanostructures characterized by two spatially distinct domains with different compositions or surface properties, offer a unique form of multifunctionality by spatially separating chemical or biological functions within the same nanostructure. Au/poly(acrylic acid) Janus NPs functionalized with folic acid and loaded with 5-ALA illustrate this concept, using one domain for targeting and another for PS delivery for PDT (Montaseri and Abrahamse 2024). Additionally, Ag-Ag_2_S Janus NPs functionalized with BSA and folic acid have been explored as targeted radiosensitizers, combining colloidal stabilization, tumor recognition, and high-Z-mediated radiation enhancement (Ghaffarlou et al. 2023). These architectures may be particularly useful when targeting, stabilization, and therapeutic activation require different surface chemistries that would be difficult to optimize on a uniform NP surface.

Hybridization with catalytic metals represents another promising strategy for improving the biological performance of photon-responsive platforms. In this context, Pt has been explored as a nanozyme due to its enzyme-mimicking activities, including catalase- and peroxidase-like properties, which can increase intracellular O_2_ availability and thereby enhance PDT-mediated ROS generation. For example, Au nanotetrapods have been synthesized through a seed-mediated growth method and subsequently conjugated with PtNPs and the PS IR808 (Zhang et al. 2024). The synergistic interaction among these components resulted in strong antitumor efficacy by integrating PTT, PDT, and catalytic therapy. Similarly, spherical AuNPs have been coated with a Pt shell to improve photodynamic performance (Quan et al. 2024). By combining these Pt-coated AuNPs with thiol-labeled DNA strands and Ce6-modified complementary DNA, an Au nanoflare platform was developed, thereby enabling enhanced PDT efficacy by integrating catalytic activity with PS delivery.

Theranostic and image-guided platforms are expected to gain relevance because BC management depends strongly on tumor localization, imaging, margin definition, and treatment monitoring. In this context, metallic nanostructures are increasingly designed not only to enhance therapeutic effects but also to guide when, where, and how therapy is activated. For example, NBPs decorated with Pt nanocrystals and loaded with indocyanine green have been explored as multifunctional theranostic nanozymes, combining computed tomography (CT)/fluorescence imaging with synergistic PTT, PDT, and CDT (Hu et al. 2024). In addition, logic-gated, surface-enhanced Raman scattering (SERS)-based AuNP platforms have enabled biomarker-activated recognition of cancer cells in vitro, followed by targeted photothermal destruction, illustrating how imaging and molecular detection can be coupled to therapy activation (Xia et al. 2025).

Future developments may also explore integrating RT with other electromagnetic-based modalities, such as radiofrequency (RF)-induced hyperthermia, to expand the therapeutic potential of metallic nanoplatforms. In this context, AuNPs may act as multifunctional agents capable of combining radiation enhancement and thermal sensitization, as shown by PEGylated AuNPs, which improved the response of MCF-7 breast cancer cells to RT combined with RF hyperthermia (Mohammadi et al. 2023).

Overall, the next generation of metallic NPs for photon-based BC therapy will likely depend on integrated design rather than on metal selection alone. Hydrogels, hybrid platforms, Janus architectures, and theranostic systems may expand the therapeutic potential of Au and AgNPs, provided that their increasing complexity is matched by reproducible synthesis, robust characterization, and clinically meaningful validation. However, the growing sophistication of these nanoplatforms should be carefully balanced with translational feasibility, ensuring that multifunctionality translates into practical and clinically applicable therapeutic solutions.

## Final remarks

The studies reviewed herein demonstrate the remarkable versatility of AuNPs and AgNPs as platforms for photon-based therapies in BC, spanning photothermal, photodynamic, radiotherapeutic, and bimodal applications. Across these modalities, therapeutic efficacy depended not only on the intrinsic physical properties of the nanomaterials but also on complex biological factors, including tumor cell characteristics, cellular uptake, subcellular localization, oxidative stress, and, in specific contexts, immune responses and DNA damage repair. These findings underscore that successful NP design requires a comprehensive understanding of both physicochemical and biological determinants of treatment response. However, translating these observations into generalizable mechanistic relationships remains challenging, as NP-mediated responses arise from multiple variables that are difficult to isolate experimentally. Differences in NP characteristics, PS properties, irradiation parameters, tumor subtype, and experimental models can influence treatment outcomes. Moreover, incomplete reporting of relevant parameters further complicates direct comparisons across studies and makes it difficult to distinguish direct NP-mediated effects from downstream biological responses. Thus, the current limitation is not necessarily a lack of mechanistic hypotheses, but rather the difficulty of validating and generalizing them across heterogeneous nanosystems and biological contexts.

Additionally, across the reviewed studies, NP performance was determined by the combined effects of particle size, surface chemistry, and functionalization, rather than by a single design parameter. Surface modifications served distinct purposes, including improving colloidal stability and biocompatibility, promoting cellular uptake or targeting, facilitating PS delivery, and modulating therapeutic responses. However, the available evidence does not support a universally superior particle size, concentration, or surface modification strategy. Instead, their relative effects depended on the therapeutic modality, biological model, and exposure conditions. Importantly, greater cellular accumulation did not necessarily translate into improved therapeutic efficacy, emphasizing that intracellular localization and biological interactions may be more relevant than uptake alone. These observations support a context-dependent approach to NP design, in which physicochemical optimization should be guided by the intended therapeutic mechanism rather than by maximizing any single parameter.

The reviewed studies also reveal important differences among photon-based modalities. While PDT has shown promising results, most investigations remain limited to in vitro models. In contrast, PTT- and RT-based applications appear to be supported by in vivo evidence, suggesting a more advanced stage of preclinical development. Nevertheless, significant challenges remain before clinical translation can be achieved for any of these approaches, as studies of all approaches exhibit substantial variability across NP designs, irradiation parameters, and/or biological systems.

These observations also raise an important question about the extent to which increasing nanosystem complexity is justified at this stage of the field. Although increasing nanosystem complexity may provide functional advantages, such complexity should be justified by demonstrable therapeutic or translational benefits. Therefore, greater complexity should not be considered an objective in itself. In our view, developing metallic nanoplatforms is most justified when each additional functional component provides a demonstrable therapeutic or translational advantage over simpler formulations. At present, the clinical potential of Au- and Ag-based nanoplatforms for photon therapies in BC remains primarily preclinical, and the available evidence does not yet support whether increasingly multifunctional systems will necessarily provide superior clinical outcomes.

Another notable finding is the predominance of AuNP-based platforms throughout the literature. Although AgNPs have demonstrated promising phototherapeutic and radiosensitizing properties, particularly through ROS-mediated mechanisms, their application remains comparatively limited, especially in advanced targeted and multimodal nanosystems. This imbalance suggests that AgNP-based platforms represent an underexplored area with considerable opportunities for future investigation.

The emergence of bimodal nanosystems further illustrates the field’s evolution toward integrated therapeutic strategies. By combining photodynamic, photothermal, and radiation-mediated mechanisms within a single platform, these systems can simultaneously target multiple cellular vulnerabilities and frequently achieve synergistic antitumor effects. Such approaches may be particularly valuable for overcoming therapeutic resistance and addressing the biological heterogeneity that characterizes BC.

Beyond the heterogeneity of NP formulations and biological models, the incomplete reporting of optical dosimetry parameters remains a significant limitation in phototherapies. In PDT and PTT studies, key experimental details, including irradiance, spot size, treatment geometry, and/or the distance between the light source and the biological target, are either partially described or omitted. This lack of methodological standardization hampers direct comparisons between studies, compromises experimental reproducibility, and limits the establishment of reliable dose–response relationships. Greater adherence to standardized reporting practices will therefore be essential for improving the quality and translational relevance of future investigations.

Although metallic NP-assisted photon therapies remain largely preclinical, AuNP-based photothermal enhancers have advanced to clinical evaluation in oncology, including ongoing trials for melanoma (ClinicalTrials.gov 2026). In contrast, clinical evidence supporting AuNP- or AgNP-mediated PDT, PTT, or RT in BC has not yet been reported, and current advances remain predominantly preclinical, particularly in vitro models.

Despite the encouraging progress observed over the past decade, major translational barriers remain. Therefore, future efforts should prioritize protocol standardization, comprehensive safety assessment, long-term biodistribution studies, and rigorous validation in clinically relevant animal models. Addressing these challenges will be essential for transforming the promising preclinical results achieved with Au and Ag nanoplatforms into safe, reproducible, and clinically effective photon-based therapies for BC.

Ultimately, the convergence of nanotechnology and photon-based therapies offers a unique opportunity to develop increasingly precise, multifunctional, and personalized treatment strategies, potentially redefining the future landscape of BC management.

## Data Availability

No datasets were generated or analysed during the current study.
